# Supplementary material for: In vivo acoustoelectric neural recording in mice enabled by ultrasound-induced frequency mixing
Source: Commun Eng. 2026 Feb 10;5:37. doi: 10.1038/s44172-026-00598-4 (PMC12920806; doi:10.1038/s44172-026-00598-4)
Supplement: Supplementary file 2 — Supplementary Information [file 44172_2026_598_MOESM2_ESM.pdf]

Supplementary Information
In vivo acoustoelectric neural recording in mice enabled by ultrasound-induced frequency mixing

Jean L. Rintoul[1*], Jonathan Howard[1], Patrycja Dzialecka[1], Xiaoqi Zhu[1], Nir Grossman[1*]

[1]Department of Brain Sciences, Imperial College London, London, W10 OHS, UK,

*Corresponding authors: j.rintoul19@alumni.imperial.ac.uk; nirg@imperial.ac.uk

SUPPLEMENTARY INFORMATION

Supplementary Note 1: In vivo electrophysiological instrumentation

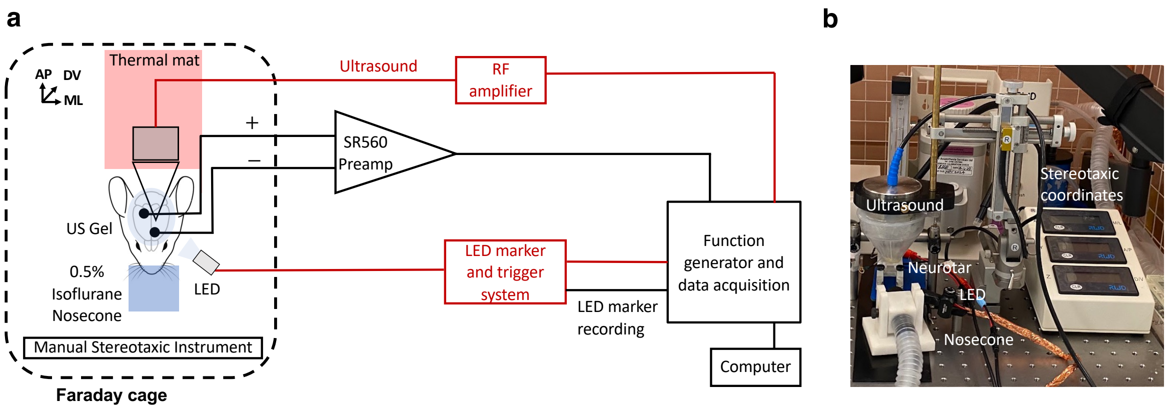

Figure S1 | Acoustoelectric instrumentation a, Acoustoelectric neural recording instrumentation for *in vivo* recording of visual evoked potentials triggered by and raspberry PI controlled LED and marker recording system, while concurrent continuous 500khz ultrasound stimulation is applied. Mouse is positioned within a Faraday Cage with a nose cone to administer Isoflurane anaesthesia, and the ultrasound positioned over the mouse head with the stereotaxic instrument which provides precise location coordinates. **b,** Photograph of electrophysiology arrangement sans mouse.

Supplementary Note 2: Electrode configuration and impedances

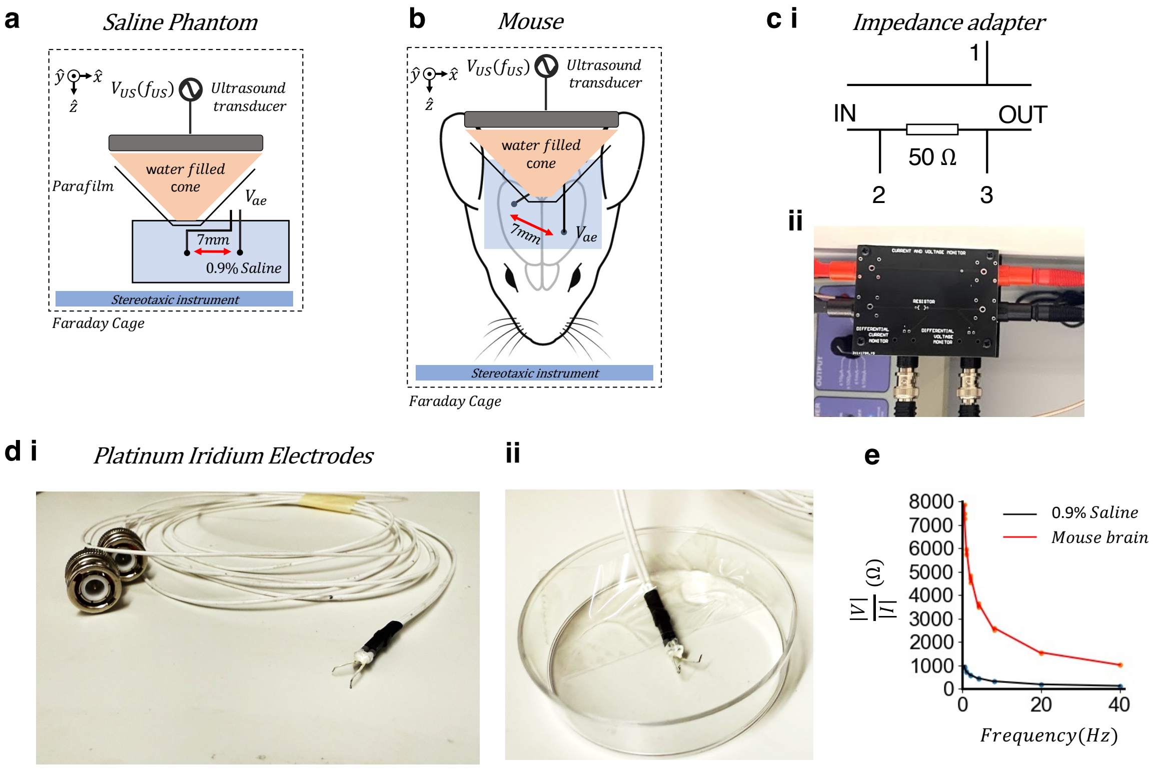

Figure S2 | Electrode configuration and impedances. Characterisation of electrode impedances, showing arrangement in both the **a,** 0.9% saline phantom and **b,** mouse brain using the same pair of 0.25mm diameter platinum- iridium electrodes spaced 7mm apart. The electrodes are insulated by white nail polish, except for 2mm of exposed tip. **b**, i) Circuit diagram of the impedance adapter. ii) Impedance

adapter photo used for $\left|\frac{V}{I}\right|$ impedance estimation. To monitor the current and voltage simultaneously an impedance adapter based on a 50 Ohm resistor was developed, to enable isolated differential measurements of the voltage (measured between 1 and 3) and the current (by measuring the potential across 2 and 3 and dividing by the known resistor value). **d**, i) Photo of the cable used for both saline phantom and mouse brain measurements, with removable 1.27mm 2 pin header which has the platinum-iridium electrode assembly which was either installed into the mouse according to the surgery described in Methods, or ii) used for saline phantom acoustoelectric testing within the electrophysiology apparatus. **e,** $\left|\frac{V}{I}\right|$ with respect to frequency calculated via impedance adapter shown in **c,** in both saline phantom and mouse described in **a** and **b.**

Supplementary Note 3: Acoustic and acoustoelectric cone characterization

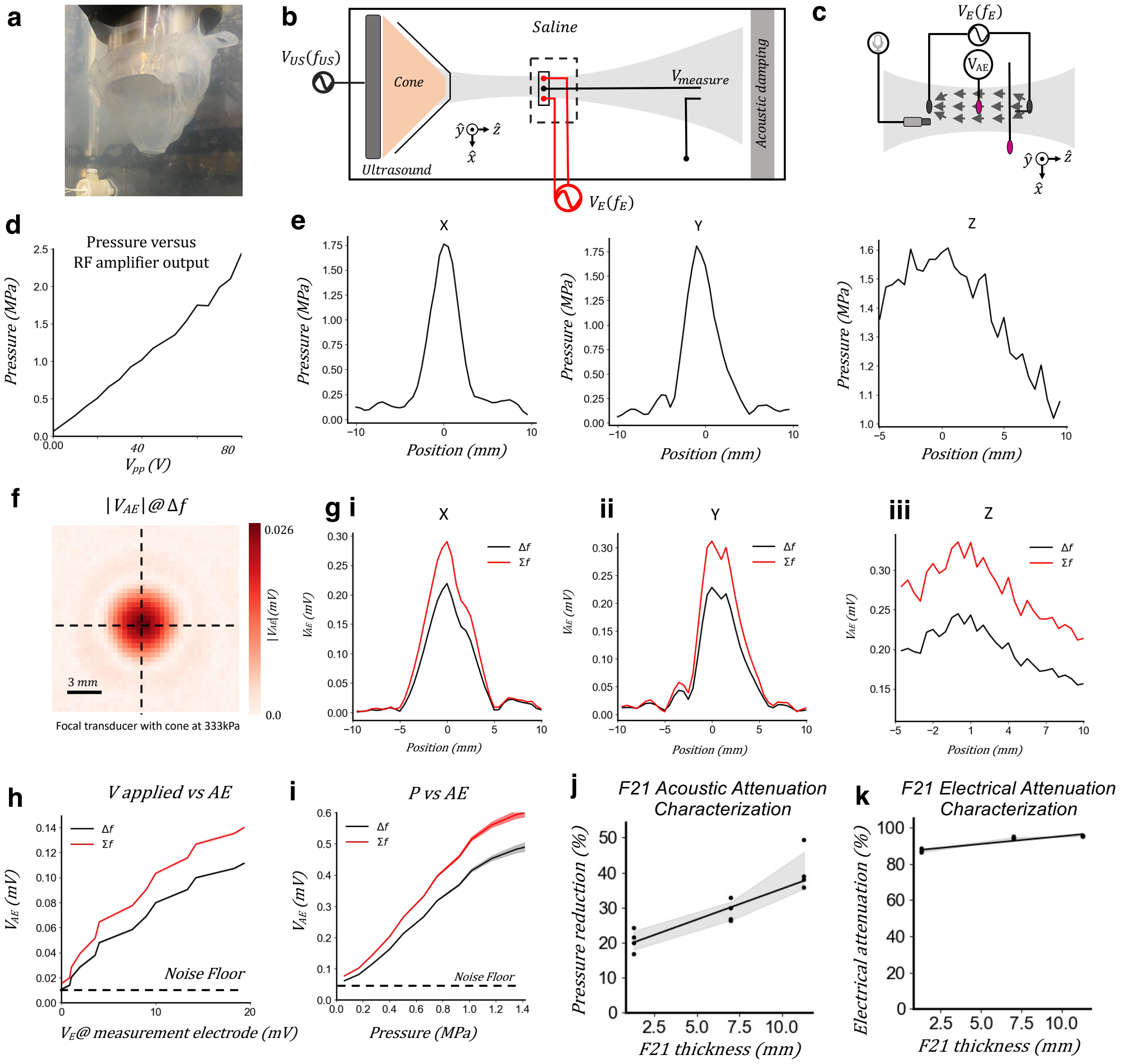

Figure S3 | Acoustic and acoustoelectric cone characterization. **a,** 500kHz ultrasound transducer with cone (70mm diameter at the top, 30mm at the bottom, 50mm height), covered in parafilm. **b,** Phantom tank (20cmx20cmx50cm) for XYZ scanning of electric fields generated through the acoustic focal area. **c,** hydrophone and electrical measurement electrodes for measuring acoustoelectric effect. **d,** Output voltage of the E&I 240L RF amplifier versus output pressure measured at the focal spot of the ultrasound transducer in 0.9% saline with hydrophone. **e,** XYZ mapping of the acoustic amplitude measured with a hydrophone. Z depth axis is made irregular by reflections off the back of the tank, despite the use of acoustic damping material. **f,** XY spatial distribution map of acoustoelectric difference frequency generated with 500kHz pressure at 333kPa, and 20Vpp@8kHz on voltage electrodes aligned with the ultrasound. 0.1s duration at 5MHz sampling rate, for each pixel. Data band filtered around

difference frequency of 492kHz. **g,** Similar parameters to f, except showing X, Y and Z axis sum (508kHz) and difference (492kHz) frequencies and their distribution for each axis. **h,** Ramping amplitude of applied 8kHz voltage with 500kHz pressure constant amplitude at 0.4MPa. Shown is sum and difference voltage amplitudes calibrated to the focal point of the transducer, with S.D. error bars over 4 recordings. 8kHz was chosen, so that a shorter time length of recording could be used in each measurement compared to an electric field at a similar frequency to the acoustic signal. **i,** Ramping pressure of 500kHz ultrasound transducer with constant 12V output 8kHz voltage on electrodes. Shown are the sum and difference voltage amplitudes calibrated to the focal point of the transducer and S.D. over 4 recordings. **j**, F21 material acoustic attenuation versus thickness. 8 second duration recordings were chosen to mimic the continuous field applied in experiments, 5MHz sampling rate repeated 4 times at each F21 thickness to generate S.D. error bar. Regression line: Attenuation (%) = 1.75 * thickness (mm) + 17.80 **k**, F21 material electrical attenuation versus thickness, 4 recordings at each point, shaded region showing S.D. error bar. Regression line: Attenuation (%) = 0.86*F21 thickness (mm) + 86.00.

For further characterization and description of the phantom XYZ stage please see the Methods and Supplemental material reported in a the previously reported physics characterization article[1].

Supplementary Note 4: Acoustoelectric independence from vibration

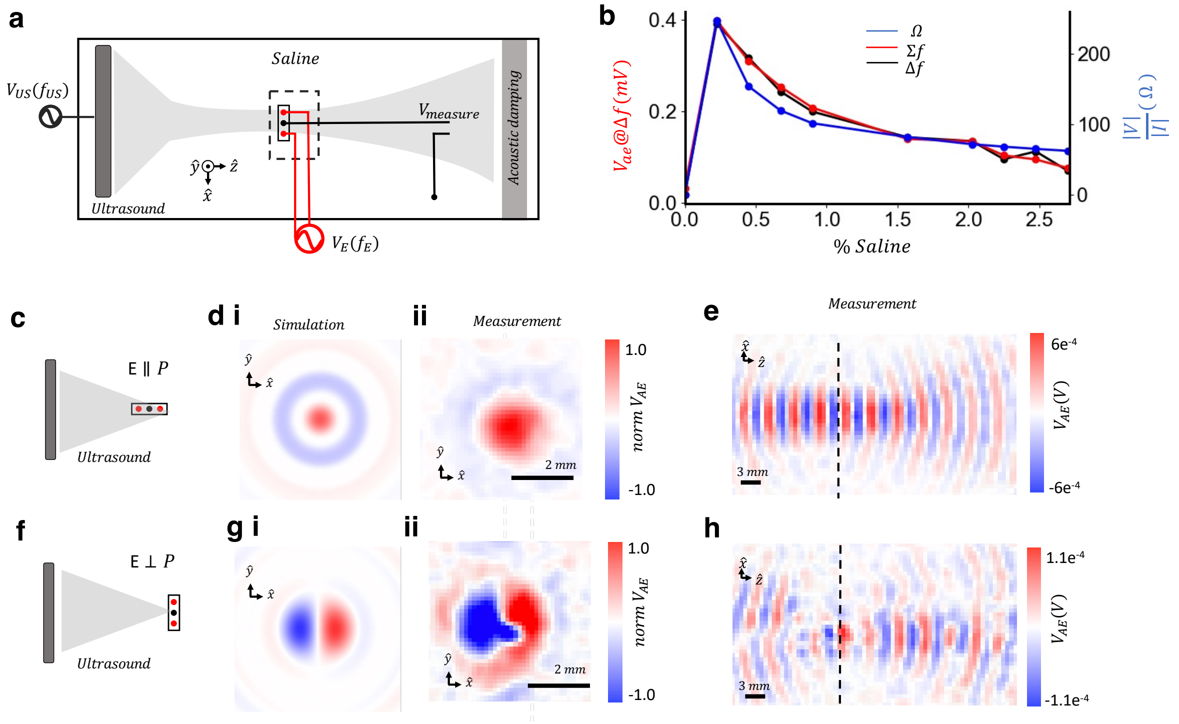

Figure S4 | Acoustoelectric independence from vibration. a, Phantom tank for XYZ scanning of electric fields based on a modified delta printer with 0.5mm movement resolution, able to calibrate to the acoustic focal maximum using a crosshair technique, with stimulation (red) platinum-iridium electrodes 7mm apart and measurement (black) electrodes, using a 500kHz ultrasound transducer. **b,** $\left|\frac{V}{I}\right|$ versus salinity, calculated by recording V and I over a 50 Ohm resistor in series with the saline load, with 10kHz 24Vpp electric field and acoustic focal maximum at 1MPa, shown in comparison with acoustoelectric field amplitude as salinity increases such that $V_{ae}(mV)$ is shown at $\Delta f =$ 490kHz, $\Sigma f = 510\text{khz}$, with each measurement repeated n=20 times as NaCl was incrementally added to de-ionized water. **c,** Spatiotemporal characteristics of the electric field generated by simultaneous application of a focal acoustic pressure field $P(x,y,z)$ at a frequency f_A=500 kHz propagating in the $\hat{z}$ direction and an electric field $\vec{E}(x,y,z)$ at a frequency f_E=8 kHz. Measurement setup same as in **a**, but with the measurement probe scanned across the volume. **c-e**, The applied electric field is parallel to the propagation direction $\hat{z}$ of the applied acoustic field. **d**, i) k-space homogeneous simulation modelling

the acoustoelectric equation[2] to show predicted radial plane $\hat{x}\hat{y}$ acoustoelectric distribution. ii) Spatial distribution of the measured electric potential V_{AE} (normalized to max value) in the radial plane $\hat{x}\hat{y}$ at the black dashed line in (**e**) **e**, Spatial distribution of the measured electric potential V_{AE} in the axial plane $\hat{x}\hat{z}$. **f-h**, The applied electric field is perpendicular to the propagation direction $\hat{z}$ of the applied acoustic field, shown are as in **c-e**.

The implanted electrodes may vibrate when exposed to the 500kHz acoustic field. If there is a vibration based electrochemical effect that induces frequency mixing, this may induce a confound to reported acoustoelectric measurements. Though this vibration based electrochemical effect is not yet reported in the literature, we can isolate the electrodes from the medium via two experiments performed in the free-field ultrasound phantom (**Fig S4 a**). The first experiment using the same electrode configuration as described in **Supplemental Note 3** except without the cone installed, shows that the acoustoelectric amplitudes can be changed by varying the salinity of the medium (**Fig S4 b**) while the electrodes remained constant, suggesting that the medium is the cause of the acoustoelectric sum and difference frequency and not the vibration at the electrodes.

To further investigate vibration confounds at the electrodes, we analysed prior free-field phantom experiments which confirm the angular dependence between the electric and acoustic field (**Fig S4 c-h**), using the same electrodes with the same spacing in each test. The orientation dependence between the two applied fields and spatial predictions of the acoustoelectric field are dependent on the acoustoelectric equation[1], and would not be present should the effect be purely based on vibration induced electrochemical effects. As a final piece of evidence based on prior work at the University of Arizona on cardiac activation mapping[3], the measurement electrode was kept in the same position and the ultrasound was moved to create a electrical cardiac activation image, also suggesting that the measured cardiac image is not due to vibrations at the electrode-tissue interface, as this would not induce a variant cardiac signal spatial map.

Supplementary Note 5: Identifying the source of the RF electrical artefact at 500kHz

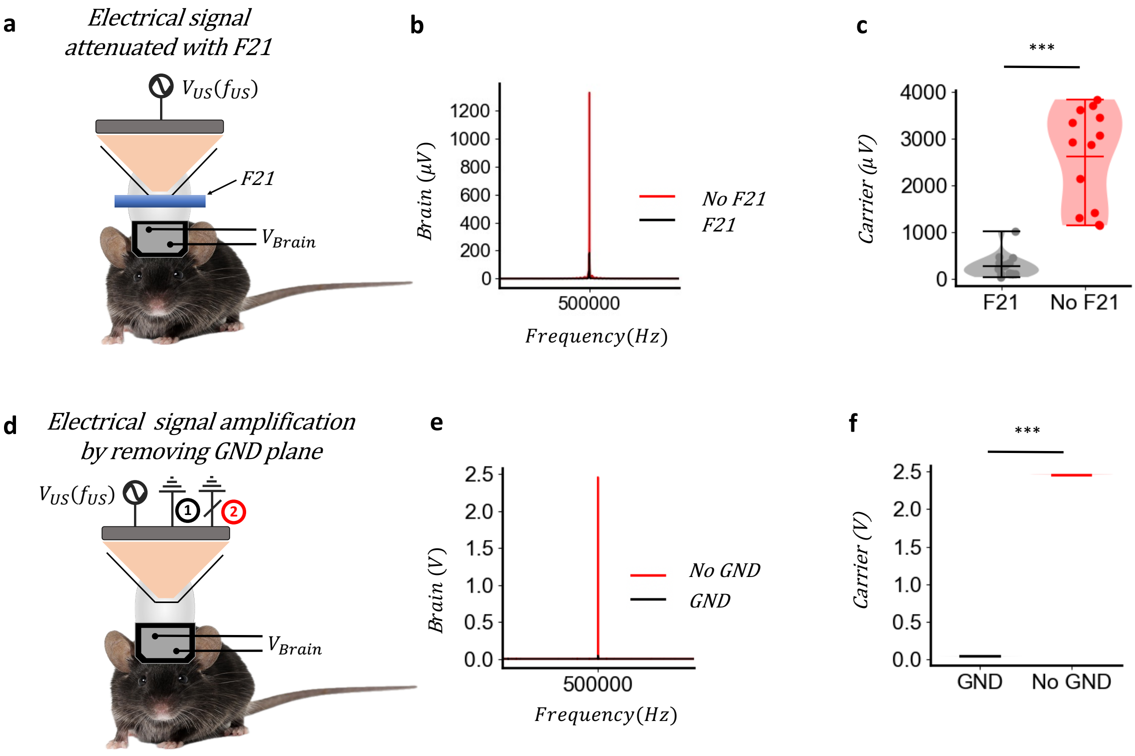

Figure S5 | Identifying the source of the electrical artefact. a, In vivo experiment arrangement for electrically attenuation and acoustic transparency with 2mm thick F21. **b,** Representative acoustic carrier amplitude ASD comparison between F21/no F21 is placed between the transducer cone and the mouse. **c,** Carrier amplitude comparison, where each mouse was tested with and without F21, with each recording of 6 second duration, using a total of 17 trials over 4 mice. t-test, two sided $t_{(16)}$= -4.99, P = 1.86e-5; F21 group (mean$\pm$s.d. = $2003.26 \pm 1410.04\mu V$); without F21 group (mean$\pm$s.d. = $270.45 \pm 219.23\ \mu V$). **d,** In vivo electric signal amplification experiment removing the ground plane of the ultrasound transducer using a switch installed into the cable. **e,** Representative ASD of large voltage 500kHz artefact when ground plane is removed. **f,** Carrier amplitude comparison with GND connected and disconnected; t-test, two sided $t_{(4)}$= -1207.06, P = 1.47e-22; No GND group (mean$\pm$s.d. = $2.46 \pm 3.90 mV$); GND group (mean$\pm$s.d. = $0.04 \pm 0.40\ mV$).

In all experiments an electric artefact was measured at the acoustic frequency, despite the separation of the water filled ultrasound cone from the subject through non-conductive parafilm. To determine the origin of this electric artefact, an electrically insulating and acoustically transparent material called F21 was used to attenuate the electric field (Precision acoustics, UK, characterized in **Fig S3 j,k**). A 500kHz sinusoid, with a 40Vpp 1MPa continuous signal was applied to the mouse, and the electric signals measured when 2mm thick F21 was inserted between the mouse and the cone, and when no material was used (**Fig S5 a, b, c**). There was a difference in the carrier amplitude when this electric shielding test was repeated over 17, 6 second trials (P = 1.86e-5). Conversely, removing the ground connection of the ultrasound transducer, by adding a switch on the transducer cable to disconnected ground while sending through the same 1MPa continuous signal, amplified the electrical transmission measured in the mouse brain as more of the energy is transmitted through the air and into the environment when the ground plane was not available as a circuit return (**Fig S5 d, e, f**). Capacitive coupling between ionic mediums enabled a high frequency electric field to be transmitted[4].

Supplementary Note 6: Anatomy of visual evoked potentials

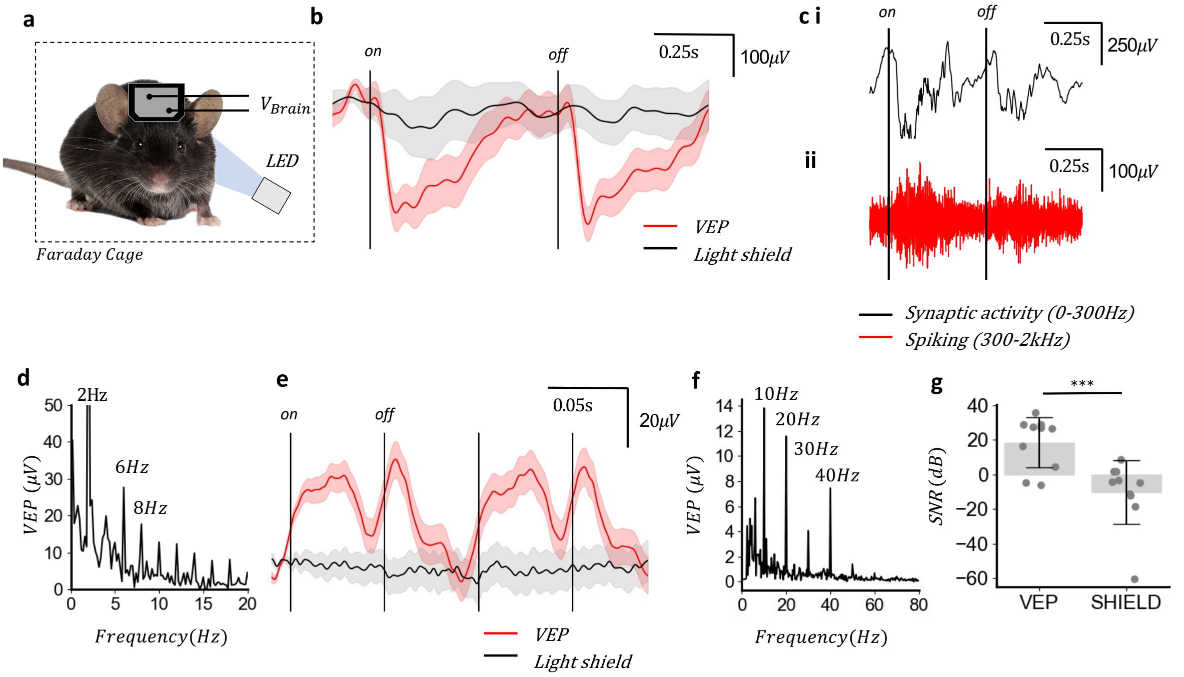

Figure S6 | Measurement and artefact test of the visual evoked potential. a, i) In vivo experimental arrangement for measuring visual evoked potentials with contralateral green LED stimulation; duration 8s, Fs = 2MHz, Preamplifier gain = 500. **b,** Single cycle of 1Hz LED showing visual evoked potential when green LED flashes (red), and when green LED flashes behind aluminium foil shielding screen (grey), data filtered 0-20Hz, n=300 events. Error bar of 95% confidence interval. **c,** Spontaneous neural activity showing the same duration as in b, with i) synaptic activity (filtered 0-300Hz) and ii) multiunit spiking activity (filtered 300Hz-2kHz). **d,** Fourier transform amplitude spectral density (ASD) of 8s duration recording averaged over 10 repeated trials of 1Hz VEP. **e,** Two ON/OFF cycles of neural signal recorded from 10Hz LED showing visual evoked potential when green LED flashes (red), and when green LED flashes behind aluminium foil shielding screen (grey), data filtered 0-20Hz, n=300 events. Error bar of 95% confidence interval. **f,** Fourier transform amplitude spectral density (ASD) of 8s duration recording averaged over 10 repeated trials of 10Hz VEP. **g,** 10 30 second trials, with signal-to-noise ratio (dB) calculated with signal being the fundamental VEP frequency(10Hz) and noise the mean of the surrounding ±2Hz. t-test, two sided $t_{(9)}$= 3.71, P = 0.001; VEP group (mean±s.d. = $18.54 \pm 14.32dB$); Shield group (mean±s.d. = = -10.82 $\pm 18.21dB$); n=10 trials.

We ensured we could measure a visual evoked potential (VEP) that was free from electrical artefacts by the pulsing a green LED at 1Hz (duty cycle 50%) while the mouse was under light anesthesia 0.5% (vol/vol) Isoflurane in Oxygen (**Fig S6 a**). A light shield was placed between the mouse and the LED, while all the same signals were applied, and we found we could only measure the electrophysiological VEP signal when the LED light was visible to the mouse (**Fig S6 b**), providing evidence that the measured VEP is of electrophysiological origins and not an electrical artefact from pulsing the LED. Furthermore, the time lag between the LED ON time and the peak deviation indicates a neural response instead of an instantaneous electrical response. The visual evoked potential measured via electrophysiology is described as under 300Hz where synaptic activity is dominant (**Fig S6 c i**), and spiking activity as between 300-2kHz[5]. Spike bursts at the peak deviations can be seen in spontaneous neural activity without averaging (**Fig S6 c ii**). The amplitude spectral density (ASD) of the VEP has a typical [6] response to both the ON and OFF conditions leading to a spectral maximum at 2Hz (**Fig S6 d**), with typical harmonics (4, 6, 8, 10Hz) continuing up the frequency spectrum at increasingly smaller amplitudes from the fundamental. More harmonics become visible as the signal to

noise increases as the duration of recording increases[7], and the thermal 1/f noise decreased. The amplitude and shape of VEPs varied between mice and duration under anesthesia.

The shield test was repeated successfully at 10Hz as this frequency is used in most experiments (**Fig S6 e**), yielding smaller peak to peak amplitudes than the 1Hz VEP. At 10Hz the neural response did not have time to recover to baseline between repeated flashes, such that the fundamental frequency had maximum amplitude (**Fig S6 f**) as compared to the other VEP harmonics. The VEP shield test was repeated over 10, 30 second trials showing a clear difference between tests (**Fig S6 g;** $t_{(9)}$= 3.71, P = 0.001).

Supplementary Note 7: DC offset is not due to neural response

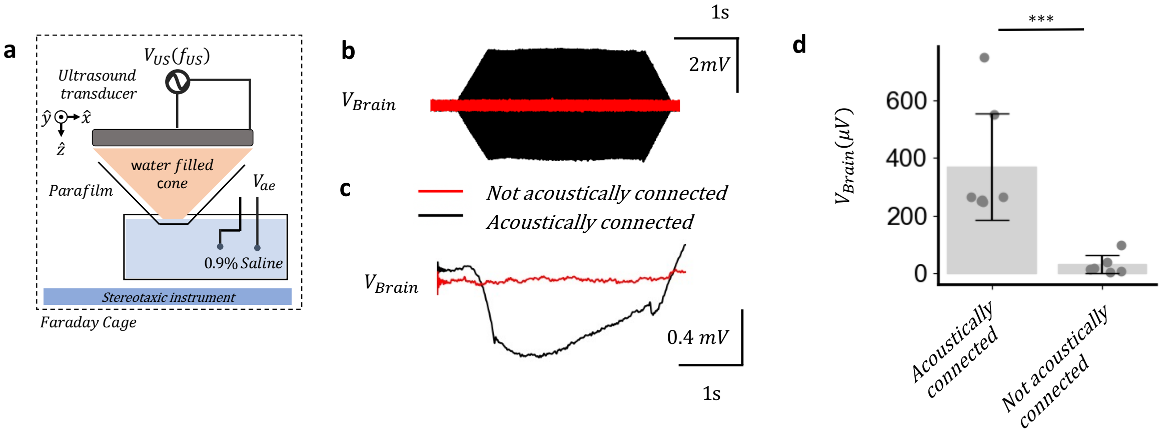

Figure S7 | Saline continuous ultrasound test to show DC offset is not due to neural response. **a,** Acoustic connection test, with 4 second recordings of 500kHz sinusoid @1MPa. Direct acoustic connection to saline, compared with air gap between saline and end of the transducer cone. **b,** Raw signal as measured in the brain. **c,** Low pass filtered V_{Brain} below 40Hz to reveal DC offset when acoustic connection to saline enables acoustic field propagation into medium. **d,** t-test comparison between groups, two sided; $t_{(6)}$= 4.06, P = 0.001; Acoustically connected (mean±sd = $369.92 \pm 185.45\mu V$); Not acoustically connected (mean±sd = = $31.55 \pm 31.62\mu V$); n=7 trials.

Supplementary Note 8: Negative spectral specificity result showing how broadband VEP harmonics are a source of artefactual demodulation

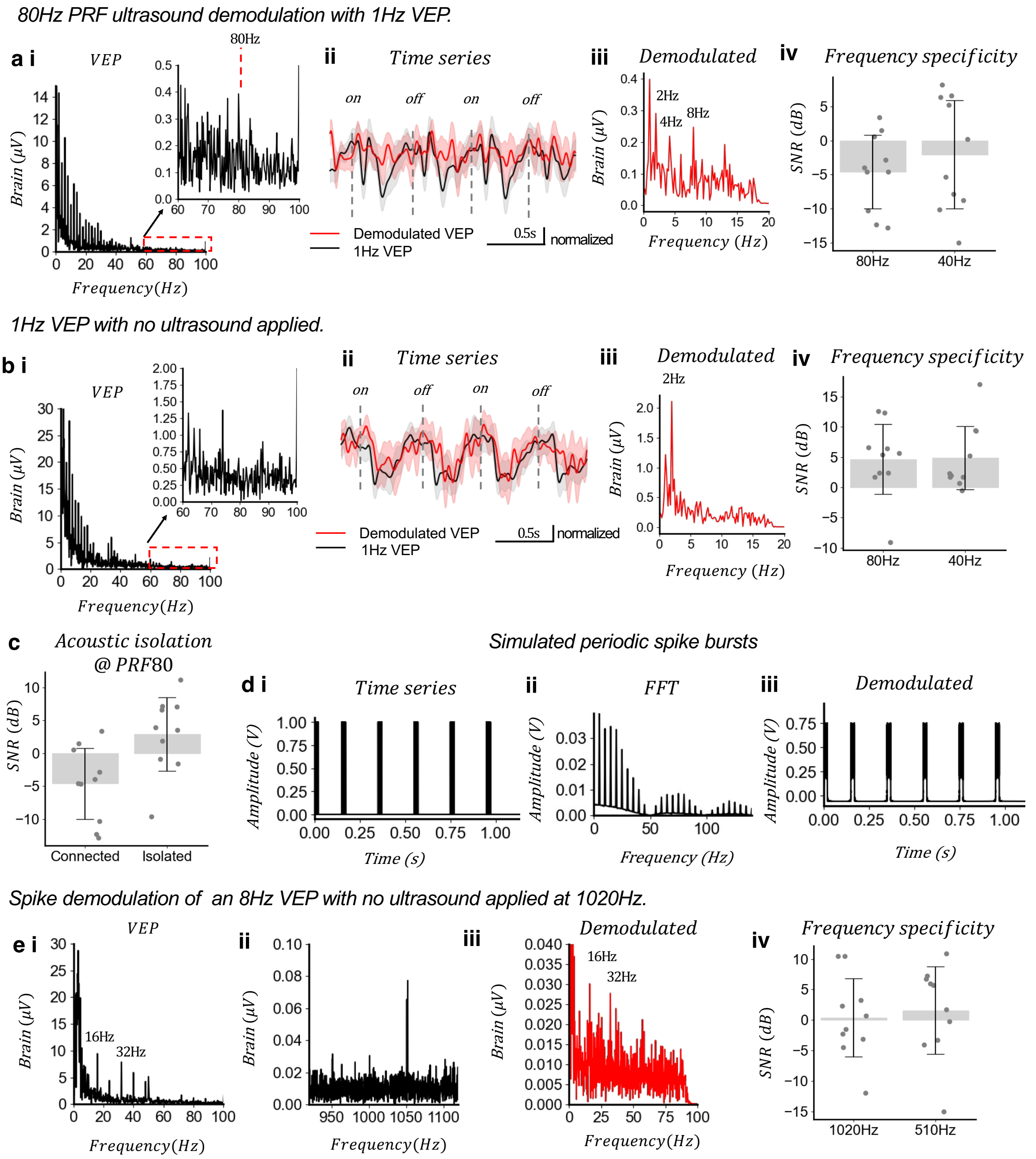

Figure S8 | Negative result spectral specificity test showing how broadband VEP harmonics are a source of artefactual demodulation. a, i), 500kHz acoustic wave pulsed at 80Hz with 50% duty cycle while a 1Hz flashing LED induces a visual evoked potential at 2,4,8Hz and upwards harmonics. Inset shows zoom view of demodulation bandwidth around 80Hz. Fs = 2MHz, duration = 8 seconds, preamplifier gain = 500 ii), IQ demodulation is performed over the $\pm 20Hz$ around the 80Hz PRF carrier frequency with 2 LED cycles shown of original VEP (black line) averaged over 20 trials with shaded region showing 95% CI and demodulated signal (red line). iii), ASD of demodulated signal shows peaks at the VEP harmonics. iv), Frequency specificity test with 10 trials in each group for n=1 mouse. t-test, two sided $t_{(9)}$= -0.79, P = 0.43; 80Hz carrier group (mean$\pm$s.d. = -4.59$\pm$5.39); 40Hz carrier group (mean$\pm$s.d. = -2.06$\pm$7.95). SNR calculated as signal = carrier $\pm 2Hz$, Noise = mean of $\pm 2Hz$ band around signal of interest. SNR = $20log_{10}(\frac{signal}{noise})$. **b,** same test as in **a**, with no ultrasound applied at all.

I) VEP harmonics ii) demodulated VEP iii) ASD of demodulated signal shows large fundamental like original signal. iv) Frequency specificity test with 10 trials in each group for n=1 mouse. t-test, two sided $t_{(9)}$= -0.09, P = 0.92; 80Hz carrier group (mean$\pm$s.d. = -4.66± 5.81); 40Hz carrier group (mean$\pm$s.d. = 4.89± 5.22). **c,** Acoustic isolation test at PRF 80Hz with 10 trials in each group for n=1 mouse. t-test, two sided $t_{(9)}$= -2.91, P = 0.009; acoustically connected (mean$\pm$s.d. = -4.59± 5.39); acoustically isolated (mean$\pm$s.d. = 2.92± 5.59). **d, i)** Simulated spike bursts formed from delta function, ii) ASD of periodic spike bursts show broadband periodic noise. Iii) IQ demodulation performed similarly to in vivo tests recovers the original spike burst signal as the delta spikes are broadband so can be demodulated from any location in the spectrum. **e,** In vivo test with 8Hz VEP, and no ultrasound, demodulating around a 1020Hz carrier. I) Original VEP and harmonics ii) spectral space to be demodulated around 1020Hz. iii) Demodulated ASD shows peaks at the VEP fundamental (16Hz) and first harmonic (32Hz) iv) Frequency specificity test with 10 trials in each group for n=1 mouse. t-test, two sided $t_{(9)}$= -0.37, P = 0.71; 1020Hz carrier group (mean$\pm$s.d. = -0.38± 6.43); 510Hz carrier group (mean$\pm$s.d. = 1.58± 7.17).

To determine if acoustoelectric heterodyning was the mechanism behind reported results of acoustoelectric brain imaging at PRF 80Hz[8–10], we pulsed the 500kHz acoustic signal at a repetition frequency of 80Hz, 50% duty cycle and demodulated a 1Hz visual evoked potential (**Fig S8 a i**) around the 80Hz carrier (**Fig S8 a ii**), recovering the original VEP harmonics through the Hilbert envelope demodulation (**Fig S8 a iii**). Though the VEP harmonics are recovered through the Hilbert envelope signal reconstruction, the frequency specificity test does not achieve a significant difference between the intended 80Hz carrier and the randomized 40Hz carrier test ($t_{(9)}$= -0.79, P = 0.43), nor can the sum and difference frequencies be seen in the modulated spectrum. This provides evidence that the demodulated signal is not dependent on a heterodyne process such as the acoustoelectric interaction.

Repeating this test without ultrasound (**Fig S8 b i,ii**), we obtain a similar successful demodulation of VEP harmonics (**Fig S8 b iii**). This result cannot be due to acoustoelectric heterodyning as no acoustic waveform is present. Once again, the spectral specificity test fails to find significant sum and difference frequencies around the carrier compared when compared to another part of the spectrum (**Fig S8 b iv**). Furthermore, the acoustic isolation test also does not achieve significance (**Fig S8 c**) providing evidence that the demodulated results are not due to acoustoelectric heterodyning.

To understand why Hilbert envelope demodulation can recover the original VEP components, we simulated periodic spike bursts (**Fig S8 d i**) made up of groups of delta signals, based on our observation of both synaptic activity and spike bursts (**Fig S8 c**). The amplitude spectral density of spike bursts decreases with frequency yet shows a sustained broadband periodic pattern (**Fig S8 d ii**) which can be demodulated to recreate a signal similar to the original signal (**Fig S8 d iii**) at any point in the spectrum. Hence spike bursts become a likely candidate for the periodic broadband noise which enables demodulation to take place at any location in the spectrum providing the amplitude of the delta signals is above the noise floor. Since the noise floor decreases with frequency (1/f) due to Poisson noise[11,12], greater sensitivity to these periodic broadband signals can be detected up to 1020Hz (**Fig S8 e i-iv**).

Previous published results on acoustoelectric brain imaging state that the mechanism behind the demodulation finding was not well-understood [8–10,13]. Unless demodulation results include a spectral specificity and acoustic isolation test proving dependence on both a heterodyne phenomenon and dependence on the presence of the acoustic signal, the periodic broadband signature of the VEP cannot be ruled out as the mechanism.

Supplementary Note 9: Two-tone electrical artefact test

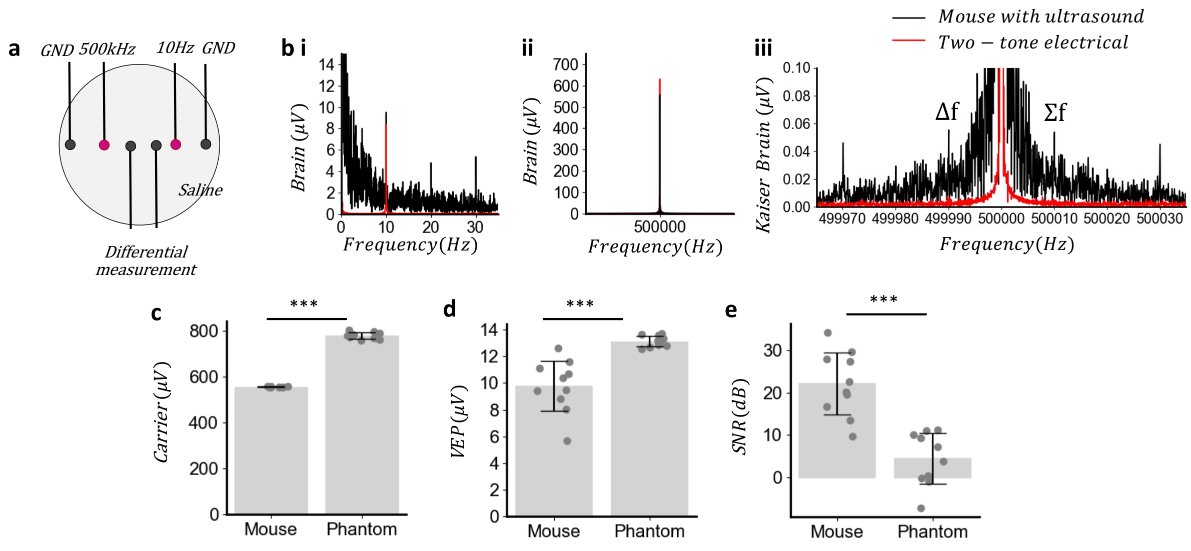

Figure S9 | Two-tone artefact test. a, Electrode arrangement in 0.9% saline phantom for two-tone signal generation test. **b,** Two-tone electrical test with 10Hz signal at similar amplitude to the visual evoked potentials seen in the experiments to show dependence on the acoustic field with preamplifier gain = 5000. i) Representative 10Hz sine wave applied in saline phantom at similar amplitude to visual evoked potential evoked *in vivo* ii) Carrier electrical artefact from ultrasound transducer (black) compared with electrical sinusoid applied at 500kHz (red). iii) Two-tone mixing test around the carrier shows modulation products only when the acoustic waveform is applied. **c,** Comparison of 500kHz carrier amplitude, shows saline phantom two-tone amplitude slightly higher than amplitude measured in a mouse from electrical carrier artefact. $t_{(9)}$= -50.03, P = 8.95e-21; Acoustically connected (mean±s.d. = $555.82 \pm 0.61\mu V$); Acoustically isolated (mean±s.d. = $779.11 \pm 13.32\ \mu V$); n=10 trials in each group. **d,** 10Hz amplitude comparison between mouse visual evoked potentials and artificially generated 10Hz sinusoid in saline phantom. t-test, two sided; $t_{(9)}$= -5.21, P = 5.83e-5; Acoustically connected (mean±s.d. = $9.78 \pm 1.87\mu V$); Acoustically isolated (mean±s.d. = $13.11 \pm 0.39\ \mu V$); n=10 trials in each group. **e,** Signal-to-noise ratio (dB) of 10Hz heterodyne products around carrier frequency compared between mouse and phantom with applied electrical carrier and 10Hz sinusoid amplitudes shown in i) and ii). t-test, two sided; $t_{(9)}$= 5.62, P = 2.46e-5; Acoustically connected (mean±s.d. = $22.12 \pm 7.31 dB$); Acoustically isolated (mean±s.d. = $4.42 \pm 5.98\ dB$); n=10 trials in each group.

Supplementary Note 10: Signal-to-noise advantages of high frequency electric signals

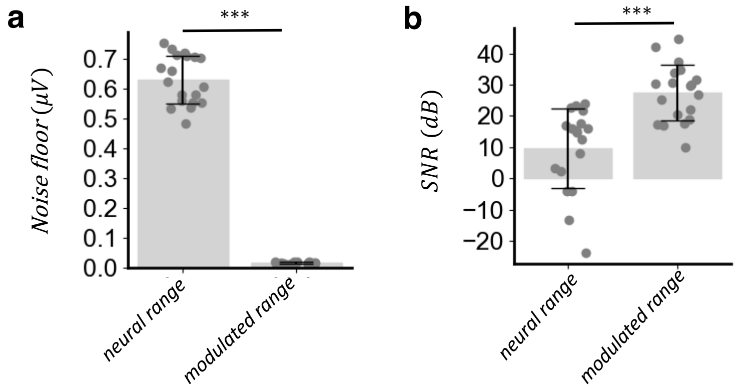

Figure S10 | Signal-to-noise comparison of the neural signal to the heterodyned products. a, Noise floor comparison in neural range (5-25Hz), compared with noise floor in modulated range

(500kHz $\pm$ 5-25Hz), calculated as mean of all spectral bins excluding the 8Hz signal bin. Noise floor amplitude comparison. t-test, two-sided; $t_{(19)}$= 32.59, P = 2.54e-28; neural range noise floor (mean$\pm$s.d. = $0.632 \pm 0.080 \mu V$); modulated noise floor (mean$\pm$s.d. = 0.015 $\pm$ 0.003 μV); n=20 trials in a single mouse. **b,** signal-to-noise ratio comparison using the noise floor calculated in a, with the signal amplitude at the 8Hz, or 500kHz $\pm$8Hz spectral bins. T-test, two-sided; $t_{(19)}$= -4.81, P = 2.71e-5; neural range SNR (mean$\pm$s.d. = $9.653 \pm 12.80\ dB$); modulated range SNR (mean$\pm$s.d. = $27.36 \pm 8.97\ dB$);

A key advantage of acoustoelectric neural recording is that shifting neural signals to high frequencies reduces thermal noise and improves signal-to-noise ratio (SNR). Using the 8 Hz in vivo dataset (**Fig. 4**), we compared the noise floor in the neural band (5–25 Hz) with the modulated band (500 kHz ± 5–25 Hz) across twenty 30s trials. Noise was significantly higher at low frequencies (0.632 ± 0.080 μV) than in the modulated range (0.015 ± 0.003 μV; **Fig. S10 a**; $t_{(19)}$= –4.81, P = 2.71e-5). Consistently, SNR was lower in the neural band (9.65 ± 12.80 dB) than at the modulated frequencies (27.36 ± 8.97 dB), corresponding to an approximately 50-fold improvement (**Fig. S10 b**). Thus, although acoustoelectric conversion efficiency is small, high-frequency modulation confers a substantial SNR benefit. Whether this advantage persists through the human skull remains to be tested.

Supplementary Note 11: Focality limitations in the mouse model

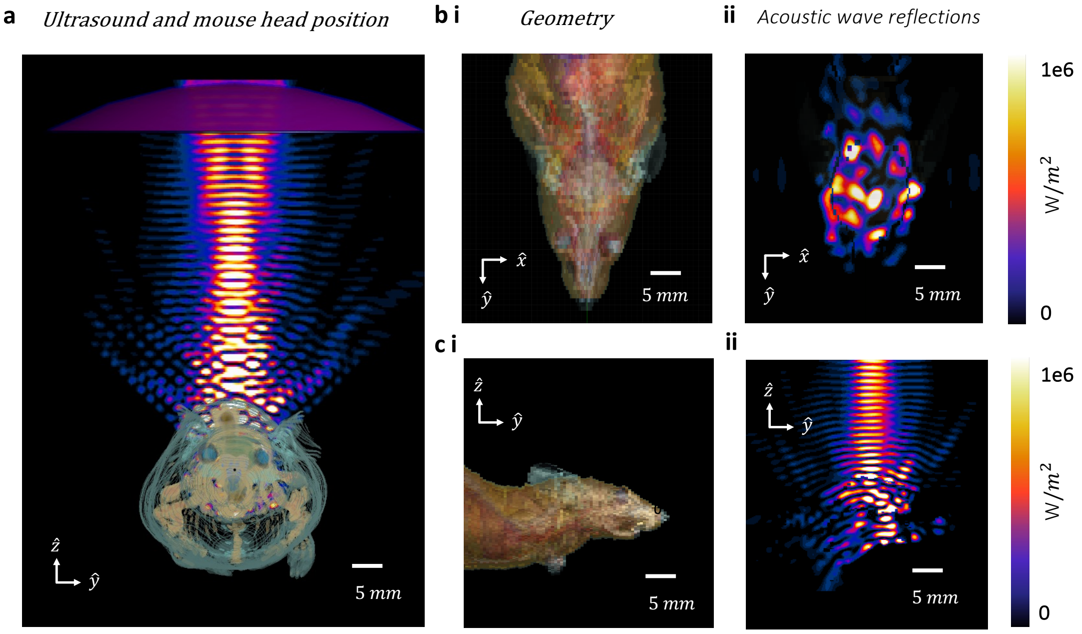

Figure S11 | Acoustic standing wave reflections. a, Geometric inhomogeneous mouse model used in Sim4Life finite element modelling simulations with 500kHz ultrasound transducer parameters matching our physical transducer experiments. **b,** i) $\hat{x}\hat{y}$ view of inhomogeneous mouse surrounded by air. ii) 100 acoustic cycles showing intensity distribution scattered throughout the mouse head. **c,** i) $\hat{x}\hat{z}$ view of inhomogeneous mouse model surrounded by air. ii) 100 acoustic cycles showing how cigar shaped focal area scatters through mouse head.

Supplementary Note 12: Temperature change over time

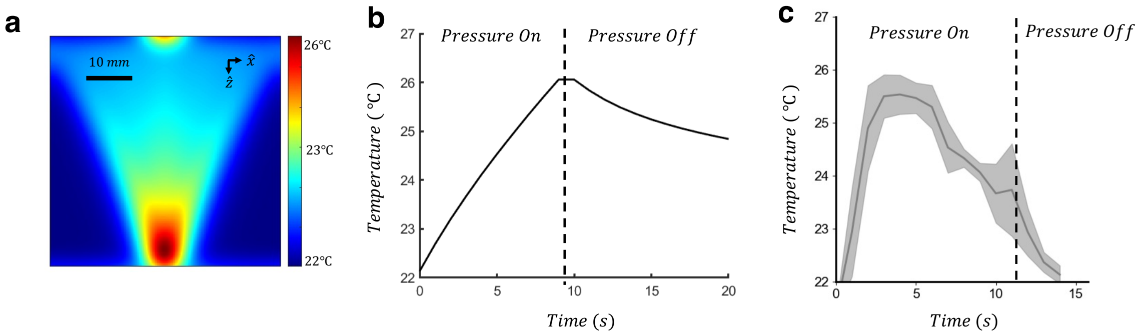

Figure S12 | Temperature monitoring. Characterisation of pressure induced thermal increase, with continuous acoustic pulse ending at the dashed line. **a**, Simulation of thermal changes using k-wave[14], showing temperature map **b,** Simulation over time, using k-wave of temperature dissipation at the ultrasound focus, with 10 second continuous pressure wave and maximum focal pressure 1 MPa **c,** Physiological phantom measurements of thermal changes at the ultrasound focus where the pressure is on from 0-10 seconds, with shaded line showing S.D. over 6 repeated measures.

A temperature probe was placed at the ultrasound focus and the change in temperature over time measured when a continuous 500kHz continuous ultrasound signal was applied with maxima at 1MPa both in simulation (**Fig S12 a, b**) and in the saline tissue phantom (**Fig S12 c**). There was a sharp onset rise when pressure onset occurs, then thermal dissipation while continuous ultrasound persists for 10 seconds (**Fig S12 c**). The thermal index[15] was exceeded within the first 1 second of continuous acoustic exposure.

SUPPLEMENTARY REFERENCES

1. Rintoul, J. L., Neufeld, E., Butler, C., Cleveland, R. O. & Grossman, N. Remote focused encoding and decoding of electric fields through acoustoelectric heterodyning. *Communications Physics 2023 6:1* **6**, 1–11 (2023).

2. Rintoul, J. L. Acoustoelectric k-space simulation and data viewing tools. Preprint at https://doi.org/10.1038/s42005-023-01198-w (2021).

3. Alvarez, A., Preston, C., Trujillo, T. & Witte, R. S. Acoustoelectric imaging for beat-to-beat cardiac activation wave mapping in an in vivo swine model. *IEEE International Ultrasonics Symposium, IUS* **2020-September**, (2020).

4. Motovilova, E. & Huang, S. Y. A review on reconfigurable liquid dielectric antennas. *Materials* **13**, (2020).

5. Buzsáki, G., Anastassiou, C. A. & Koch, C. The origin of extracellular fields and currents — EEG, ECoG, LFP and spikes. *Nature Reviews Neuroscience 2012 13:6* **13**, 407–420 (2012).

6. Ridder, W. H. & Nusinowitz, S. The visual evoked potential in the mouse—Origins and response characteristics. *Vision Res* **46**, 902–913 (2006).

7. Rapuano, S. & Harris, F. J. An introduction to FFT and time domain windows. *IEEE Instrum Meas Mag* **10**, 32–44 (2007).

8. Song, X., Su, X., Chen, X., Xu, M. & Ming, D. In Vivo Transcranial Acoustoelectric Brain Imaging of Different Steady-State Visual Stimulation Paradigms. *IEEE Transactions on Neural Systems and Rehabilitation Engineering* **30**, 2233–2241 (2022).

9. Song, X., Chen, X., Guo, J., Xu, M. & Ming, D. Living Rat SSVEP Mapping With Acoustoelectric Brain Imaging. *IEEE Trans Biomed Eng* **69**, 75–82 (2022).

10. Zhou, Y. *et al.* In Vivo Transcranial Acoustoelectric Brain Imaging of Different Deep Brain Stimulation Currents. *IEEE Transactions on Neural Systems and Rehabilitation Engineering* 1–1 (2024) doi:10.1109/TNSRE.2024.3356440.

11. Gilden, D. L., Thornton, T. & Mallon, M. W. 1/f Noise in Human Cognition. *Science (1979)* **67**, 1837–1839 (1995).

12. Gerster, M. *et al.* Separating Neural Oscillations from Aperiodic 1/f Activity: Challenges and Recommendations. *Neuroinformatics* **20**, 991–1012 (2022).

13. Zhou, Y. *et al.* Coding Biological Current Source with Pulsed Ultrasound for Acoustoelectric Brain Imaging: Application to Vivo Rat Brain. *IEEE Access* **8**, 29586–29594 (2020).

14. Treeby, B. E. & Cox, B. T. k-Wave: MATLAB toolbox for the simulation and reconstruction of photoacoustic wave fields. *J Biomed Opt* **15**, 021314 (2010).

15. Duck, F. A. The Meaning of Thermal Index (TI) and Mechanical Index (MI) Values. *Ultrasound* **5**, 36–40 (1997).
