# Supplementary material for: In vivo acoustoelectric neural recording in mice enabled by ultrasound-induced frequency mixing
Source: Commun Eng. 2026 Feb 10;5:37. doi: 10.1038/s44172-026-00598-4 (PMC12920806; doi:10.1038/s44172-026-00598-4)
Supplement: Supplementary file 3 — Reporting Summary [file 44172_2026_598_MOESM3_ESM.pdf]

nature portfolio

Corresponding author(s): Jean Rintoul

Last updated by author(s): Dec 25, 2025

Reporting Summary

Nature Portfolio wishes to improve the reproducibility of the work that we publish. This form provides structure for consistency and transparency in reporting. For further information on Nature Portfolio policies, see our Editorial Policies and the Editorial Policy Checklist.

Statistics

For all statistical analyses, confirm that the following items are present in the figure legend, table legend, main text, or Methods section.

n/a	Confirmed	
☐	☒	The exact sample size (n) for each experimental group/condition, given as a discrete number and unit of measurement
☐	☒	A statement on whether measurements were taken from distinct samples or whether the same sample was measured repeatedly
☐	☒	The statistical test(s) used AND whether they are one- or two-sided<br>*Only common tests should be described solely by name; describe more complex techniques in the Methods section.*
☐	☒	A description of all covariates tested
☐	☒	A description of any assumptions or corrections, such as tests of normality and adjustment for multiple comparisons
☐	☒	A full description of the statistical parameters including central tendency (e.g. means) or other basic estimates (e.g. regression coefficient) AND variation (e.g. standard deviation) or associated estimates of uncertainty (e.g. confidence intervals)
☐	☒	For null hypothesis testing, the test statistic (e.g. F, t, r) with confidence intervals, effect sizes, degrees of freedom and P value noted<br>*Give P values as exact values whenever suitable.*
☒	☐	For Bayesian analysis, information on the choice of priors and Markov chain Monte Carlo settings
☒	☐	For hierarchical and complex designs, identification of the appropriate level for tests and full reporting of outcomes
☐	☒	Estimates of effect sizes (e.g. Cohen's d, Pearson's r), indicating how they were calculated

Our web collection on statistics for biologists contains articles on many of the points above.

Software and code

Policy information about availability of computer code

Data collection	The applied voltages, monitor channels and electric potentials were logged using data acquisition hardware (WiFiScope WS5, WiFiScope WS6 DIFF and Handyscope HS5, TiePie engineering, Netherlands), and its API used to control recording parameters.
Data analysis	Custom Python scripts were created to using the numerical analysis coding libraries NumPy, Pandas and SciPy libraries.

For manuscripts utilizing custom algorithms or software that are central to the research but not yet described in published literature, software must be made available to editors and reviewers. We strongly encourage code deposition in a community repository (e.g. GitHub). See the Nature Portfolio guidelines for submitting code & software for further information.

Data

Policy information about availability of data

All manuscripts must include a data availability statement. This statement should provide the following information, where applicable:

- Accession codes, unique identifiers, or web links for publicly available datasets
- A description of any restrictions on data availability
- For clinical datasets or third party data, please ensure that the statement adheres to our policy

The data supporting the results in this study are available within the paper and its supplementary information. The raw and analysed data-sets generated during the study are available for research purposes from the corresponding authors on reasonable request. A representative example of single-shot demodulation is available on figshare DOI: https://figshare.com/s/2ca17d7ca823aaa59748.

Research involving human participants, their data, or biological material

Policy information about studies with human participants or human data. See also policy information about sex, gender (identity/presentation), and sexual orientation and race, ethnicity and racism.

Reporting on sex and gender	No humans were used in this study.
Reporting on race, ethnicity, or other socially relevant groupings	No socially relevant groupings were used as no humans were used in this study.
Population characteristics	No humans were used in this study.
Recruitment	No recruiting was required as no humans were used in this study.
Ethics oversight	Human ethics approval was not relevant for this study as it does not involve humans.

Note that full information on the approval of the study protocol must also be provided in the manuscript.

Field-specific reporting

Please select the one below that is the best fit for your research. If you are not sure, read the appropriate sections before making your selection.

☒ Life sciences ☐ Behavioural & social sciences ☐ Ecological, evolutionary & environmental sciences

For a reference copy of the document with all sections, see nature.com/documents/nr-reporting-summary-flat.pdf

Life sciences study design

All studies must disclose on these points even when the disclosure is negative.

Sample size	The sample size was determined based on initial pilot studies and the laboratories prior work and similar approaches in the field.
Data exclusions	No data was excluded from the analyses.
Replication	Data were replicated as indicated in the figures and Methods. For all animal studies, replicates were performed on different days as independent experiments.
Randomization	Animals from different cages and gender were selected to assure randomization.
Blinding	Blinding was not required in this study, as it tests the engineering efficacy of a new physics based neuroimaging technique and not differences between animal cohorts.

Reporting for specific materials, systems and methods

We require information from authors about some types of materials, experimental systems and methods used in many studies. Here, indicate whether each material, system or method listed is relevant to your study. If you are not sure if a list item applies to your research, read the appropriate section before selecting a response.

Materials & experimental systems

n/a	Involved in the study	
☒	☐	Antibodies
☒	☐	Eukaryotic cell lines
☒	☐	Palaeontology and archaeology
☐	☒	Animals and other organisms
☒	☐	Clinical data
☒	☐	Dual use research of concern
☒	☐	Plants

Methods

n/a	Involved in the study	
☒	☐	ChIP-seq
☒	☐	Flow cytometry
☒	☐	MRI-based neuroimaging

Animals and other research organisms

Policy information about studies involving animals; ARRIVE guidelines recommended for reporting animal research, and Sex and Gender in Research

Laboratory animals	All mice were wild type C57BL/6 male and female mice, aged between 3-6 months, purchased from Charles River Laboratories Ltd.
Wild animals	The study did not involve wild animals.
Reporting on sex	Equal numbers of male and female mice were used.
Field-collected samples	The study did not involve samples collected from the field.
Ethics oversight	The Imperial College of London's Animal Welfare and Ethical Review Body approved all animal procedures, and all experiments were performed in accordance with relevant regulations/according to the United Kingdom Animals (Scientific Procedures) Act 1986.

Note that full information on the approval of the study protocol must also be provided in the manuscript.

Plants

Seed stocks	The study did not involve seed stocks.
Novel plant genotypes	The study did not involve plant genotypes
Authentication	The study did not involve plants so authentication of seed stocks was not required.
